# Lipid profiling of mouse intestinal organoids for studying *APC* mutations

**DOI:** 10.1042/BSR20202915

**Published:** 2021-03-17

**Authors:** Zoë Jukes, Anne Freier, Maria Glymenaki, Richard Brown, Lee Parry, Elizabeth Want, Panagiotis A. Vorkas, Jia V. Li

**Affiliations:** 1 Division of Digestive Diseases, Department of Metabolism, Digestion and Reproduction, Faculty of Medicine, Imperial College London, London, U.K.; 2 Division of Systems Medicine, Department of Metabolism, Digestion and Reproduction, Faculty of Medicine, Imperial College London, London, U.K.; 3 European Cancer Stem Cell Research Institute, Cardiff University, School of Biosciences, Hadyn Ellis Building, Maindy Rd, Cardiff, CF24 4HQ, U.K.; 4 Institute of Applied Biosciences, Centre for Research and Technology Hellas, 57001 Thessaloniki, Greece

**Keywords:** colorectal cancer, lipidomics, lipids, liquid chromatography-mass spectrometry, metabolomics, organoids

## Abstract

Inactivating mutations including both germline and somatic mutations in the adenomatous polyposis coli (*APC*) gene drives most familial and sporadic colorectal cancers. Understanding the metabolic implications of this mutation will aid to establish its wider impact on cellular behaviour and potentially inform clinical decisions. However, to date, alterations in lipid metabolism induced by *APC* mutations remain unclear. Intestinal organoids have gained widespread popularity in studying colorectal cancer and chemotherapies, because their 3D structure more accurately mimics an *in vivo* environment. Here, we aimed to investigate intra-cellular lipid disturbances induced by *APC* gene mutations in intestinal organoids using a reversed-phase ultra-high-performance liquid chromatography mass spectrometry (RP-UHPLC-MS)-based lipid profiling method. Lipids of the organoids grown from either wild-type (WT) or mice with *APC* mutations (Lgr5–EGFP-IRES-CreER^T2^ *Apc^fl/fl^*) were extracted and analysed using RP-UHPLC-MS. Levels of phospholipids (e.g. PC(16:0/16:0), PC(18:1/20:0), PC(38:0), PC(18:1/22:1)), ceramides (e.g. Cer(d18:0/22:0), Cer(d42:0), Cer(d18:1/24:1)) and hexosylceramides (e.g. HexCer(d18:1/16:0), HexCer(d18:1/22:0)) were higher in *Apc^fl/fl^* organoids, whereas levels of sphingomyelins (e.g. SM(d18:1/14:0), SM(d18:1/16:0)) were lower compared with WT. These observations indicate that cellular metabolism of sphingomyelin was up-regulated, resulting in the cellular accumulation of ceramides and production of HexCer due to the absence of *Apc^fl/fl^* in the organoids. Our observations demonstrated lipid profiling of organoids and provided an enhanced insight into the effects of the *APC* mutations on lipid metabolism, making for a valuable addition to screening options of the organoid lipidome.

## Introduction

Colorectal cancer is the third most common cancer and the fourth most common cause of cancer deaths worldwide [1]. Most incidences are preventable and related to unhealthy lifestyle choices such as low-fibre diets, consumption of processed meats, and obesity [2]. However, approximately 5% of cases are associated with hereditary conditions, such as familial adenomatous polyposis (FAP). The patients with FAP have one mutated copy of the *APC* gene and carry a near 100% lifetime risk of developing colorectal cancer [3]. Even in cases of sporadic colorectal cancer, loss of functional APC is typically the first mutational event to occur [4]. In fact, *APC* mutations have been found in over 80% of colonic tumours [4]. Given its prevalence, uncovering the wider effects of an inactivating *APC* mutation is important to fully understand colorectal cancers and aid the development of more effective personalised treatments. The *APC* gene encodes the tumour suppressor protein APC, which regulates the degradation of proto-oncogene protein β-catenin. β-Catenin acts as a transcriptional co-regulator of pro-proliferative signalling [5]. Wild-type (WT) APC binds β-catenin and becomes a major component in the destruction complex. The destruction complex is a multiprotein complex, which phosphorylates β-catenin, creating a binding site for an E3 ubiquitin ligase, which in turn tags it for ubiquitin-mediated degradation [5]. The exact role of APC in the destruction complex has not been fully elucidated. However, it has been shown that transiently introducing WT APC into the human colonic SW480 cell line, which expresses a truncated form, leads to a dramatic reduction in β-catenin levels [6]. Consistently, inactivating mutations in *APC* gene have been shown to be mutually exclusive of activating mutations in β-catenin in cancers [7]. Previous studies have shown that inhibition of β-catenin in the pancreas suppresses tumour growth [8]. Part of this signal suppression mechanism appears to be facilitated by the *SGMS2* gene, which is an important regulator of lipids including sphingomyelins and ceramides [9].

Many studies have highlighted complex differences in metabolic profiles of tissues from colorectal cancer patients compared with healthy individuals [10,11]. Lower levels of lipids were found in colon cancerous tissue compared with healthy mucosa [12]. However, metabolic profiling of tissue samples can be confounded by the presence of other mutations in tumours, hence any metabolic differences cannot be directly attributed to the *APC* mutation. To overcome this challenge, the current work sought to study the metabolome of organoids grown from *Apc^fl/fl^* and WT mice using reversed-phase ultra-high-performance liquid chromatography coupled to mass spectrometry (RP-UHPLC-MS)-based lipidomics approach.

An organoid is a 3D *ex vivo* multicellular culture derived from stem cells to mimic a specific organ or tissue [13]. Organoid studies provide superior opportunities for more complex modelling. This is due to their 3D nature, which provides a milieu that more closely resembles *in vivo* tissues. Organoids also more accurately mimic *in vivo* cell–cell interactions and differentiation, whereas cell lines do not generally maintain this complexity [14]. Because β-catenin and APC play an important role in cell–cell adhesions [15,16], 3D tissue structures provide a preferable model to study the implications of *APC* mutations. Furthermore, organoids provide advantages for use in metabolic studies over tissue slices. While tissue slices represent an *in vivo* environment, organoids can guarantee the purity of cell types. The latter is difficult to ensure when using tissue samples [17]. Therefore, organoid cultures allow data to be collected from specific desired cell types.

Zhang et al*.* (2018) have previously shown that lipid metabolism was perturbed in Apc^−/+^ mice [18]. In the present study, we aimed to identify *APC* mutation-induced changes in lipid metabolites by comparing the lipid profiles of small intestinal organoids derived from WT and *Apc^fl/fl^* mice. To our knowledge, this is the first study to determine the effects of *APC* mutations on lipid metabolites in mouse organoids using a highly sensitive analytical technique. The present study demonstrated an analytical pipeline for organoid metabolic profiling. The findings in the present study provide lipid biomarkers of cancerous and normal cells and enhanced insight into the metabolic effects of inactivating *APC* mutations commonly found in colorectal cancer tumours, and thus could inform therapeutic strategies in the future.

## Materials and methods

### Organoid culture

Animal experiment and culture of the mouse upper small intestinal (USI) organoids were carried out at University of Cardiff. Work was approved by a U.K. Home Office Project license (PPL30/3279; protocols 6 and 7). Animals were maintained on an outbred background and housed in a standard facility under a 12-h light cycle, with ad libitum water and diet (expanded diet, Special Diet Services U.K.). Control (*Lgr5–EGFP-IRES-CreER^T2^ Apc^+/+^*) or experimental (*Lgr5–EGFP-IRES-CreER^T2^ Apc^fl/fl^*) adult mice were administered tamoxifen (80 mg/kg) daily via intraperitoneal injection for four consecutive days to induce *Cre* expression. Fourteen days following induction mice were killed (cervical dislocation) and their intestinal cells were harvested for organoid culture of WT or *Apc* deficient intestinal stem cells [19]. Organoids were provided from male 2 wild-type (WT) (WTm1641 and WTm1663) and 2 *Apc^ΔISC/ΔISC^* (APCm830 and APCm862) donors. Organoids derived from each donor were cultured in two 24-well plates with approximately 50 organoids per well. At the point of collection, six wells were collated into one sample to ensure sufficient amount of metabolites for lipid profiling. Hence, eight samples were obtained per donor.

### Lipid metabolite extraction

Sample extraction was carried out based on a previously published protocol [20]. In brief, following aqueous extraction using cold methanol and water (v:v, 1:1), 1 ml of pre-chilled dichloromethane (DCM)/methanol (v:v, 3:1) was added to the organoid samples. Samples were bead-beaten for 40 s followed by 5 min of chilling on dry ice. This procedure was repeated three times before being centrifuged for 10 min at 21,000 rcf at 4°C. A total of 600 μl of supernatant from each sample was transferred to a glass vial. Another 200 μl of supernatant from each sample was pooled into a 15-ml Falcon tube to form a quantity control (QC) sample and split into several aliquots of 600 μl each. An extraction blank sample was included to control for any potential contaminant introduced throughout the extraction process. Samples were dried by evaporation over night at room temperature and stored at −40°C until further analysis.

### Lipid extract reconstitution

The dried extracts were reconstituted in 100 μl of water/acetonitrile (ACN)/isopropanol (IPA), (v:v:v, 1:1:3). The lipids were dissolved by vigorous vortexing for 5 min, followed by 5 min of sonication. This step was repeated three times to allow the dry extracts to thoroughly dissolve in the solvent. Samples were subsequently centrifuged at 21,000 rcf for 10 min at 4°C and transferred to 150-μl glass inserts placed in glass vials (Waters).

### UHPLC-MS data collection

For the lipid analysis, a quadrupole-time of flight (Q-TOF) MS (Waters Synapt G2-S, U.K.) coupled to an Acquity UPLC system (Waters, U.K.) was used. For chromatographic separation, the reversed-phase C18 CSH column (2.1 × 10 mm, 1.7 μm) (Waters Corp) was used at 55°C. Mobile phase A consisted of ACN/water (v:v, 3:2), 10 mM ammonium formate and 0.1% formic acid. Mobile phase B consisted of IPA/ACN (v:v, 9:1) and 0.1% formic acid. Both phases were degassed by sonication for 5 min. Column flow rate and elution gradients were set according to Vorkas et al*.* [20]. Injection volumes were set at 4 μl. MS spectra were acquired in both positive and negative electrospray ionisation (ESI) modes within a mass range of 50–2000 Da. Leucine encephalin was used as the lock mass to correct mass drifts throughout the analytical run. The system was calibrated with sodium formate before the start of the analytical run. The QC was injected 10 times at the start of both the negative and positive runs to condition the column. The run order of the samples was randomised and a QC sample was injected every eight experimental samples, followed by the QC dilution series (v:v, 1:2, 1:4, 1:8) and an extraction blank. Furthermore, data-dependent acquisition fragmentation spectra (DDAs) were acquired in both ESI modes and using QC samples, to aid with metabolite annotations.

### UHPLC-MS data processing and analysis

All data analyses were performed using R studio version 3.4.1. Pre-processing and quality control: Peak picking, grouping of features and retention time correction were carried out using XCMS release 3.7 [21]. Individual parameters were refined after inspection of the base peak intensity chromatograms. Related features were annotated using the *CAMERA* package [22]. Features with a coefficient of variation >30% in the QC samples and those with an average intensity of >70% of the blank injections were omitted from statistical analysis [23]. Data analysis: To enhance comparability, data were normalised using probabilistic quotient normalisation. Variance was stabilised using the generalised log transformation with parameter lambda estimated according to the QC samples [24]. Unit variance scaling method was used prior to multivariate statistical analysis. Principal components analysis (PCA) was carried out to reduce the number of dimensions and allow visualisation of data patterns. QC samples were assessed for tight clustering to verify instrumental stability throughout the run [25]. One outlying sample (*Apc^fl/fl^*) was removed in positive and negative ESI mode analyses.

Data were assessed using univariate (Welch’s *t*-test) and multivariate analyses. Specifically, orthogonal projections to latent structures - discriminant analysis (OPLS-DA) was performed (*ropls* package [26]) to find the greatest separation between WT and APC classes or between the donors within the same genotype. The supervised analysis aims to find the greatest separation between sample classes [27]. Features were deemed ‘important’ to the classification task if their variable importance scores were >1.5. Model performance was assessed by training the model on a random subset of the samples and then computing the predictions on the training subset and the test subset.

Top features according to the VIP cut-off were further subjected to univariate analysis (Welch’s *t*-test) to determine if they were statistically significant between the *Apc^fl/fl^* and WT groups. Resulting *P*-values were corrected for multiple comparison (Benjamini–Hochberg procedure) [28]. We considered an adjusted *P*<0.05 as statistically significant. For significant features, fold changes (FC) were calculated. FCs > 1.4 were deemed as overexpressed and those < 0.7 were considered to be underexpressed.

### Metabolite identification

Significant features (adjusted *P*<0.05) were putatively annotated using online databases [29–31] and literature [20]. Assignments were verified by inspection of the data-dependent MS/MS spectra and allocated a confidence score depending on the certainty of their structural elucidation. Scores may be interpreted as follows: (1) observed median *m/z* matched to database-reported *m/z*, additional adduct matched where possible; (2) meet criteria 1 plus adduct and at least one fragment match to databases; (3) meet criteria 1 and multiple fragments matched to databases.

### Pathway analysis

Identified lipids (scores 2 and 3) were subjected to pathway analysis using Ingenuity Pathway Analysis (IPA) software (build version 463341M, QIAGEN Bioinformatic, 2018). Canonical pathways were considered significant at *P*<0.05.

## Results

### Significant differences in lipid composition between *Apc^fl/fl^* and wild-type organoids

PCA was initially performed on the LC-MS profiles acquired in the positive and negative electrospray ionization (ESI) modes, respectively. The PCA score plots unveiled tight clustering of the QC samples, which was formed by pooling a small amount from each sample in the experiment, demonstrating satisfactory instrumental stability (Supplementary Figures S1 and S2). The QC dilution series samples approached the QCs cluster, as their concentration increased. Both PCA score plots showed the same outlier, a sample from *APC^fl/fl^* m862 donor, which was due to a sample loss during the sample extraction, thus this sample was excluded from the subsequent analyses.

**Figure 1:**
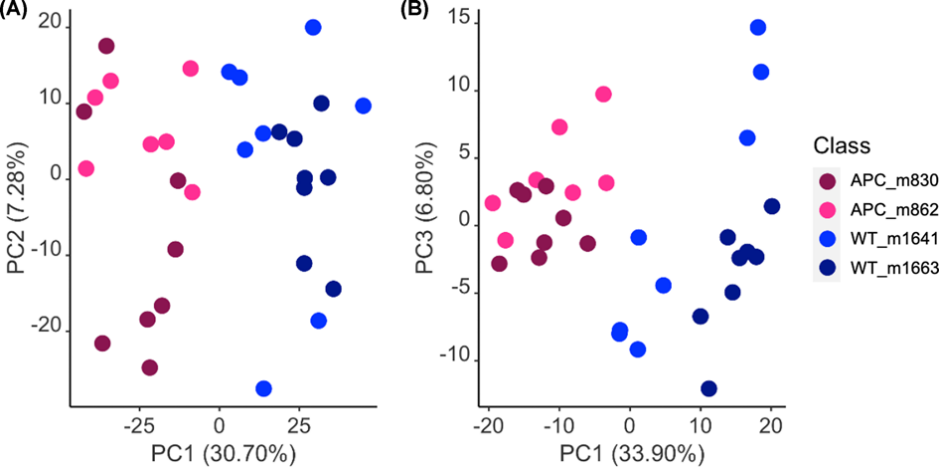
PCA scores plots of LC-MS-based lipid profiles from *Apc*^*fl/fl*^ (pink) and WT (blue) organoids PCA scores plots of LC-MS-based lipid profiles from *Apc^fl/fl^* (pink) and WT (blue) organoids in ESI+ (**A**) and ESI- (**B**) modes. The percentage on each axis represents the proportion of variance (R^2^X) explained by the principal component one, two or three (PC1, PC2 or PC3). Two shades of pink and blue colors represent the mouse donors of the organoids.

PCA was carried out based on the data from the WT and *Apc^fl/fl^* organoids and a clear separation between the two groups was observed along the first principal component in both ESI+ and ESI- modes (Figure 1 and Supplementary Figure S3). Interestingly, no distinct cluster was seen between these outbred mouse donors within the same genetic background (i.e. between WT m1641 and WT m1663), suggesting that variations contributed by *APC* mutations are more dominant than inter-donor variations from the same group.

**Figure 2:**
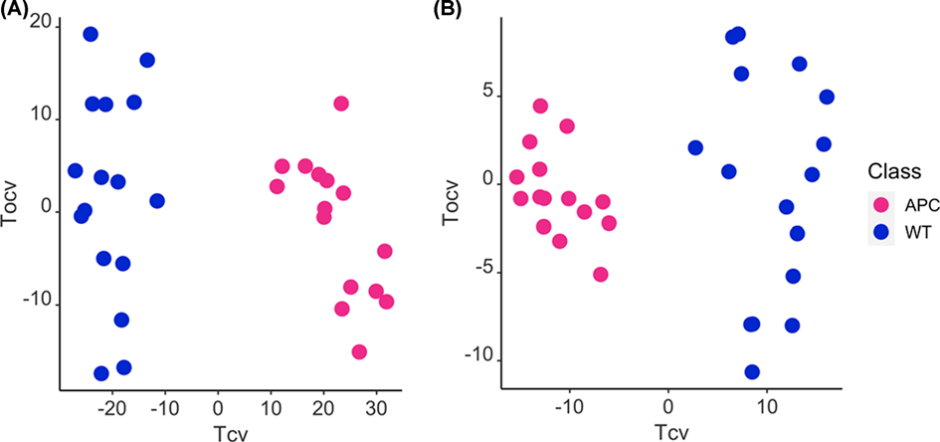
OPLS-DA cross-validated scores plots of LC-MS-based lipid profiles from *Apc ^fl/fl^* and WT organoids OPLS-DA cross-validated scores plots of LC-MS-based lipid profiles from *Apc^fl/fl^* (pink) and WT (blue) organoids in ESI+ ((**A**) R^2^X = 36.9%; Q^2^Y = 0.93; CV-ANOVA *P*=1.05 × 10^−14^) and ESI- ((**B**) R^2^X = 40.5%; Q^2^Y = 0.90; CV-ANOVA *P*=1.14 × 10^−12^) modes.

Following PCA analysis, we performed supervised analysis, orthogonal partial least squares discriminant analysis (OPLS-DA), for both ESI- and ESI+ mode. The OPLS-DA reveals a significant separation between the WT and *Apc^fl/fl^* groups, suggesting significant biochemical differences in lipid composition of the organoids between the two groups (Figure 2). Assessment of the predictive performance of the models using a training and test subset of the data resulted in excellent classification predictive performance, reflected by Q^2^Y (0.93 and 0.9 for ESI+ and ESI-, respectively). All features were further assessed using univariate statistics (Welch’s *t*-test). Features with an adjusted *P*-value < 0.05 and a VIP score > 1.5 were deemed significant and taken forward for identification: a total of 204 in ESI+ mode and 31 in ESI- mode unique features.

**Figure 3:**
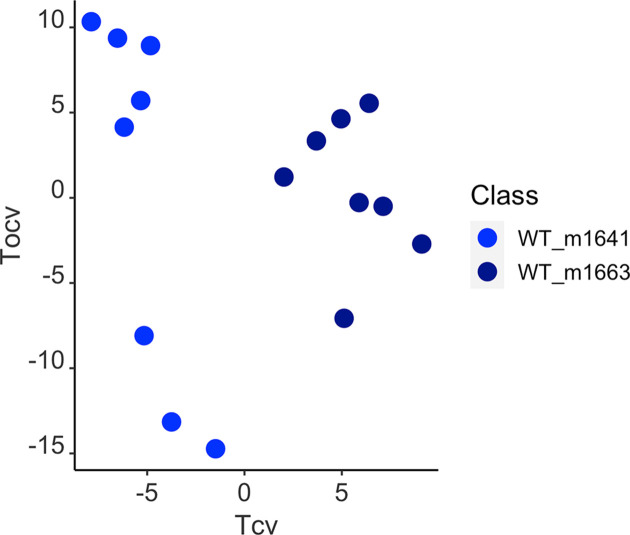
OPLS-DA cross-validated scores plot of LC-MS-based lipid profiles from two wild-type donors OPLS-DA cross-validated scores plots of LC-MS-based lipid profiles from two wild-type (WT) donors in ESI- mode (R^2^X = 38.5%; Q^2^Y = 0.80; CV-ANOVA *P*=0.0007).

Furthermore, we applied OPLS-DA to investigate lipid changes that are associated with donors to allow for an intra-class comparison. No significant changes were observed between donors in either *Apc^fl/fl^* or WT in ESI+ mode. While no significant change was seen between *Apc^fl/fl^* donors in ESI- mode, a statistically significant model was obtained between WT donors (Figure 3) and a total of nine features (Supplementary Table S1) were deemed significant.

### Wild-type and *Apc^fl/fl^* organoids show significantly different levels of sphingolipids and phospholipids

Lipids were identified by matching experimental accurate mass and MS/MS spectral data to theoretical mass and fragmentation patterns of literature [20] and online databases including Metlin [30], LipidMaps [29] and Human Metabolome Database (HMDB) [31]. The top signatures of significantly different lipids (Table 1) highlight varying intensity trends (fold change) between WT and *Apc^fl/fl^* organoids. The majority of lipid sub-classes—including ceramides and phosphatidylcholines—were found to be more abundant in the *Apc^fl/fl^* group compared with WT organoids. Specifically, dihydroceramides (e.g. Cer(d18:0/22:0)) and their direct metabolites ceramides (e.g. Cer(d18:1/23:0)) and phytoceramides (e.g. Cer(t18:0/22:0)) were found to be higher in *Apc^fl/fl^* compared with WT. Significant lipid sub-classes such as the hexosylceramides were among the most abundant. Sphingomyelins consistently exhibited lower levels in *Apc^fl/fl^* compared with the wild-type.

**Table 1:** Annotated lipids that contributed to the genotype or donor discrimination

Metabolites	Retention time (min)	Observed median (*m/z*)	PPM	Databased reported (*m/z*)	Adducts	Formula	Level of identification	Fold change
**APC vs WT (ESI+)**								
Cer(d18:1/23:0)	13.9	658.6105	0	658.6114	[M+Na]+	C41H81NO3Na	3	1.63
Cer(d18:2/24:0)	13.7	670.6108	0	670.6114	[M+Na]+	C42H81NO3Na+	3	2.35
Cer(t18:0/22:0)	13.3	640.6234	2	640.6238	[M+H]+	C40H81NO4	3	1.84
DG(18:1_18:1)	13.5	603.5330	3	603.5352	[M+H-H_2_O]+	C39H71O4+	3	2.14
DG(O-16:0_18:0)	14.0	565.5554	1	565.556	[M+H-H_2_O]+	C37H74O4	3	2.5
HexCer(d18:1/16:0)	6.3	682.5616	0	682.5622	[M+H-H_2_O]+	C40H77NO8	3	2.36
HexCer(d18:1/22:0)	13.1	806.6462	2	806.648	[M+Na]+	C46H89NO8	3	3.29
HexCer(d42:0)	13.8	836.6959	2	836.6974	[M+H]+	C50H94NO8	3	2.84
HexCer(d42:1)	13.6	834.6784	1	834.6793	[M+Na]+	C48H93NO8Na	3	4.21
HexCer(t18:1/24:0)	13.2	668.6549	5	668.6551	[M+H]+	C42H85NO4	3	26.54
lysoPC(18:0)	1.7	524.3715	1	524.3716	[M+H]+	C26H55NO7P+	3	-1.87
PC(16:0_16:0)	7.8	734.5692	0	734.5694	[M+H]+	C40H80NO8P	3	2.62
PC(18:1_18:2)	7.5	806.5666	0	806.567	[M+Na]+	C44H82NO8P	3	-2.04
PC(18:1_20:0)	12.9	816.6469	0	816.6477	[M+H]+	C46H90NO8P	3	1.74
PC(18:1_22:1)	13.0	842.6612	2	842.6633	[M+H]+	C48H92NO8P	3	2.22
PC(38:0)	13.0	840.6359	11	840.6453	[M+Na]+	C46H92NO8P	3	1.9
PC(O-22:5_16:0)	8.3	816.6080	0	816.5902	[M+H]+	C45H87NO9P	3	37.29
SM(d18:1/14:0)	4.4	697.5248	1	697.5255	[M+Na]+	C37H76N2O6P+	3	-2.12
Stearoylcarnitine	1.8	428.3739	1	428.3734	[M+H]+	C25H49NO4	3	54.82
DHCer(d18:0/22:0)	13.8	606.6179	2	606.6189	[M+H-H_2_O]+	C40H81NO3	2	2.04
Cer(d42:1)	13.4	632.6333	0	632.6346	[M+H-H_2_O]+	C42H83NO3	2	2.06
Cer(d42:1)	14.1	632.6339	1	632.6346	[M+H-H_2_O]+	C42H83NO3	2	2.8
HexCer(d42:2)	13.1	832.6626	1	832.6637	[M+Na]+	C48H91NO8Na	2	4.95
HexCer(t18:1/24:1)	13.1	850.6868	15	850.6742	[M+Na]+	C48H93NO9Na	2	3.53
PE(35:1)	13.0	732.5418	16	732.5538	[M+H]+	C40H78NO8P	2	2.99
SM(d18:1/16:0)	5.7	703.5744	1	703.5754	[M+H]+	C39H80N2O6P	2	-2.55
DHCer(d18:0/22:0)	13.6	606.6189	4	606.6184	[M+H-H_2_O]+	C40H80NO2	1	12.29
Cer(d18:1/26:0)	14.5	660.6645	2	660.6653	[M+H-H_2_O]+	C44H86NO2	1	4.54
Cer(d42:0)	14.1	634.6471	5	634.6502	[M+H-H_2_O]+	C42H85NO3	1	2.92
Cer(d42:2)	13.8	670.6105	1	670.6109	[M+Na]+	C42H81NO3	1	1.51
Cer(d42:2)	14.1	630.6152	5	630.6189	[M+H-H_2_O]+	C42H81NO3	1	3.34
Cer(d44:0)	14.3	680.6905	1	680.6915	[M+H]+	C44H89NO3	1	3.87
Cer(m18:0/24:0)	14.5	636.6654	0	636.6653	[M+H]+	C42H86NO2	1	4.37
Cer(m40:1)	13.6	628.6004	0	628.6003	[M+Na]+	C40H79NO2Na	1	2.3
Cer(t38:2)	13.5	630.5522	14	630.5432	[M+Na]+	C38H73NO4	1	1.89
diHexCer(d40:1)	12.9	968.6997	2	968.7009	[M+Na]+	C52H99NO13	1	10.81
diHexCer(d42:1)	13.5	956.7394	0	956.7402	[M+H-H_2_O]+	C54H103NO13	1	4
HexCer(d18:0/26:0)	13.9	880.7185	3	880.7212	[M+Na]+	C50H99NO9Na	1	2.1
HexCer(d18:1/26:0)	14.1	862.7097	0	862.7106	[M+Na]+	C50H97NO8	1	6.25
HexCer(d34:0)	7.0	724.5692	0	724.5697	[M+Na]+	C40H79NO8	1	4.4
PC(36:1)	9.4	752.5986	2	752.5963	[M+H-2H_2_O]+	C44H86NO8P	1	3.86
PC(O-30:0)	6.9	692.5588	2	692.5589	[M+H]+	C38H79NO7P	1	8.33
PE(O-32:1)	10.6	704.5584	0	704.5589	[M+H]+	C39H78NO7P	1	8.57
PG(O-37:1)	8.3	795.6294	0	794.6269	[M+NH_4_]+	C43H85O9P	1	28.36
**APC vs WT (ESI-)**								
PE(O-18:1_18:0)	13	730.6003 (730.5765)	1	730.5756	[M-H]-	C41H81NO7P	3	2.41
PE(42:1)	13.1	829.6888 (828.6549)	7	828.6488	[M-H]-	C47H91NO8P	3	2.75
Cer(d18:1/24:1)	13.7	692.6207	1	692.6198	[M+Formate]-	C42H81NO3	2	2.44
HexCer(d18:1/24:1)	13.1	854.7014	0	854.6726	[M+Formate]-	C48H91NO8	2	4.13
SM(d18:1/16:0)	5.8	747.5867	4	747.5658	[M+Formate]-	C39H79N2O6P	2	-2.87
Cer(d41:1)	13.1	680.6189	1	680.6199	[M+Formate]-	C42H82NO5	1	3.09
HexCer(d18:0/22:0)	13.3	830.6701	3	830.6726	[M+Formate]-	C46H91NO8	1	3.26
HexCer(d18:0/24:0)	13.8	812.6972	1	812.6985	[M-H]-	C48H95NO8	1	2.57
HexCer(d18:0/26:0)	13.7	856.7195	2	856.6883	[M+Formate]-	C48H93NO8	1	3.78
HexCer(d18:1/24:0)	13.3	856.6874	7	856.6883	[M+Formate]-	C48H93NO8	1	3.44
PI(18:0_18:1)	7.4	863.5577	5	863.5655	[M-H]-	C45H85O13P	1	2.6
**WT donors (m1641 vs m1663) (ESI-)**								
Cer(d38:1)	13	638.5721	1	638.5724	[M+Formate]-	C39H76NO5	2	1.83
HexCer(d18:0/24:0)	13.8	858.7016	7	858.7039	[M+Formate]-	C48H95NO8	2	1.73

Positive and negative fold change indicate higher or lower relative levels of lipids in *Apc^fl/fl^* organoids compared to wild-type (WT), or in WT-m1641 compared with WT-m1663. Abbreviations: Cer, ceramide; DG, diacylglycerol; DHCer, dihydroceramides; GalCer, galactosylceramide; GlcCer, glucosylceramide; HexCer, hexosylceramide; LacCer, lactosylceramide; PC, phosphatidylcholines; PE, phosphatidylethanolamines; PG, phosphatidylglycerol; PI, phosphatidylinositol; PIP, phosphatidylinositol bisphosphate; PS, phosphatidylserine; SM, sphingomyelin; Levels of annotation: (1) observed median *m/z* matched to database-reported *m/z*, additional adduct matched where possible; (2) meet criteria 1 plus adduct and at least one fragment match to databases; (3) meet criteria 1 and multiple fragments matched to databases.

Many of the significant features were found to be isotopic pairs of carbon (^12^C and ^13^C) as deduced by *CAMERA* and assessment of feature intensities in representative spectra. Due to low signal intensity and strong co-elution of lipid species in the positive mode data-dependent acquisition mode, around 40% of significant features could not be identified and these features are summarized in Supplementary Table S1.

### Differentially expressed pathways between WT and *Apc^fl/fl^* organoids

The affected canonical pathways, as predicted by ingenuity pathway analysis, are outlined in Table 2. Ceramide signalling, sphingosine-1-phosphate signalling, phosphatidylethanolamine biosynthesis II, p70S6K signalling and mTOR signalling (*P*<0.05) were found to be up-regulated in *Apc^fl/fl^*, whereas sphingomyein metabolism, RhoA signalling, Rac signalling, ceramide signalling and sphingosine-1-phosphate signalling (*P*<0.05) were down-regulated compared with WT. Notably, although some pathways (i.e. ceramide signalling and sphingosine-1-phosphate signalling) overlap, the specific lipids contributing to these pathways were different.

**Table 2:** Overview of the top five differentially expressed pathways and corresponding *P*-values by up-regulated and down-regulated lipid classes in *Apc^fl/fl^* organoids compared to wild-type

	Name of pathway	*P*-value	Lipids used to predict pathway
**Up-regulated**	Ceramide signalling	1.86E-04	Cer, DHCer
	Sphingosine-1-phosphate signalling	1.86E-04	Cer, DHCer
	Phosphatidylethanolamine biosynthesis II	1.86E-04	PE, PS
	p70S6K signalling	4.62E-04	PC, DG
	mTor signalling	4.62E-04	PC, DG
**Down-regulated**	Sphingomyelin metabolism	1.46E-05	SM, PC
	RhoA signalling	2.34E-03	PC
	Rac signalling	3.53E-03	PC
	Ceramide signalling	4.70E-03	SM
	Sphingosine-1-phosphate signalling	4.70E-03	SM

## Discussion

This work used RP-UHPLC-MS-based lipidomics to profile organoids grown from small intestine of WT and *Apc^fl/fl^* mice. The observed intensity differences in lipid classes between WT and *Apc^fl/fl^* organoids highlighted the distinct cellular characteristics associated with the *Apc^fl/fl^* phenotype. We identified an increase in phosphatidylcholines (PC) in *Apc^fl/fl^* compared with WT, which is consistent with previous studies on cancerous cells and tissues, where choline phospholipid metabolism was disturbed [32–34].

We found that levels of sphingomyelins, which are important constituents of cell membranes, were lower in *Apc^fl/fl^* than WT organoids. Sphingolipids have been shown to be reduced in patients with colorectal cancer [35], although some evidence suggested that this may not be due to *APC* knockout [36]. Sphingomyelin hydrolysis is an established pathway in the generation of ceramides [37], the higher levels of which were observed in *Apc^fl/fl^* organoids in the current study. Ceramides are currently understood to function as secondary messengers that coordinate responses to cellular stress and mediate apoptosis [38]. Various studies have shown that ceramides were accumulated under a variety of stressful conditions, such as oxidative stress, radiation and heat [39,40]. Ceramides can be produced predominantly by two biochemical pathways: sphingomyelin hydrolysis and *de novo* ceramide synthesis. The observed reduction of sphingomyelin levels indicates the activation of the former pathway for ceramide production, while an increase in dihydroceramides (DHCer) further implicates the pathway of *de novo* ceramide synthesis as an afford means for ceramide production. Importantly, DHCer themselves, as well as the downstream dihydro analogues of DH-SM and DH-HexCer, have been recently found to be implicated in several biological function including apoptosis [41], autophagy and cell proliferation [42]. Additionally, cellular levels of hexosylceramides were elevated in *Apc^fl/fl^* organoids, as represented by multiple lipid species. While further research is needed to confirm that the identified HexCers are glucosylceramides (GlcCer) or galactosylceramide (GalCer), these HexCers are more likely to be GlcCer as hypothesized by Separovic et al. [43] since galactosylceramide synthase, an enzyme catalyzing the formation of GalCer, is predominantly expressed in the kidney, testis, Schwann cells, cerebrum and cerebellum [44,45]. GlcCer are also membrane lipids and are synthesized by transferring of glucose to ceramides under catalysis of glucosylceramide synthase (GCS) [46]. GlcCer have been linked to effects distinct from ceramides. While ceramides act in a tumour-suppressive manner, ceramide glycosylation reduces the ceramide concentrations, thus resuming cellular processes to protect cancer cells [46]. Studies have shown that GCS was associated with cancer drug resistance [47–49] and this drug resistance could be selectively reversed by inhibiting GCS to reduce GlcCer production [50]. Our findings demonstrated that cellular metabolism of sphingomyelin was up-regulated, resulting in the cellular accumulation of ceramides and production of GlcCer due to the *APC* mutation in *Apc^fl/fl^* organoids.

The sphingolipid sub-class of diHexCers have a sugar dimer attached to the backbone of their sphingoid base. In the present study, diHexCer(d40:1) and diHexCer(d42:1) increased 10.8 and 4.0 -fold in *Apc^fl/fl^* organoids, respectively. Previous research found that LacCers (a diHexCer) was accumulated in multidrug resistance human sarcoma cell lines (DX5 and GARF cells), and a positive correlation between the levels of LacCers and inhibitor resistant P-glycoprotein, a multidrug resistance protein [51]. This observation supported the speculation that *APC* mutations confer drug resistance through changes in lipid metabolism, which warrant further investigation.

## Conclusion

The lipid profile of *Apc^fl/fl^* mouse intestinal organoids identified in the present study provided insights into the effects of an inactivating *APC* mutation on lipid metabolism and may guide the development of biomarkers underlying *APC* mutations. Given the agreement to previous findings [52], the present study showed that future therapeutics designed for patients with FAP could be tested in colonic organoids. The global lipid profiles of treated organoids could be used to investigate if the potential therapeutics confer a shift towards the WT profile. We believe that in combination with metabolomics analysis, this would be a powerful tool during the early stages of drug screening.

## Supplementary Material

Supplementary Figures S1-S3 and Table S1

## Data Availability

These data are available at the NIH Common Fund’s National Metabolomics Data Repository (NMDR) website, the Metabolomics Workbench, https://www.metabolomicsworkbench.org, where it has been assigned Project ID PR001092. The data can be accessed directly via its Project DOI: 10.21228/M8JM5Z.

## Open Access

Open access for this article was enabled by the participation of Imperial College London in an all-inclusive *Read & Publish* pilot with Portland Press and the Biochemical Society under a transformative agreement with JISC.

## Abbreviations

APC: adenomatous polyposis coli
Cer: ceramide
FAP: familial adenomatous polyposis
GalCer: galactosylceramide
GCS: glucosylceramide synthase
GlcCer: glucosylceramides
HexCer: hexosylceramides
LacCer: lactosylceramide
OPLS-DA: orthogonal projections to latent structures - disciminant analysis
PCA: principal component analysis
QC: quality control
SM: sphingomyelin
WT: wild-type
