# Supplementary material for: Lipid profiling of mouse intestinal organoids for studying APC mutations
Source: Biosci Rep. 2021 Mar 17;41(3):BSR20202915. doi: 10.1042/BSR20202915 (PMC7969701; doi:10.1042/BSR20202915)
Supplement: Supplementary Figures S1-S3 and Table S1 [file BSR-2020-2915_supp.pdf]

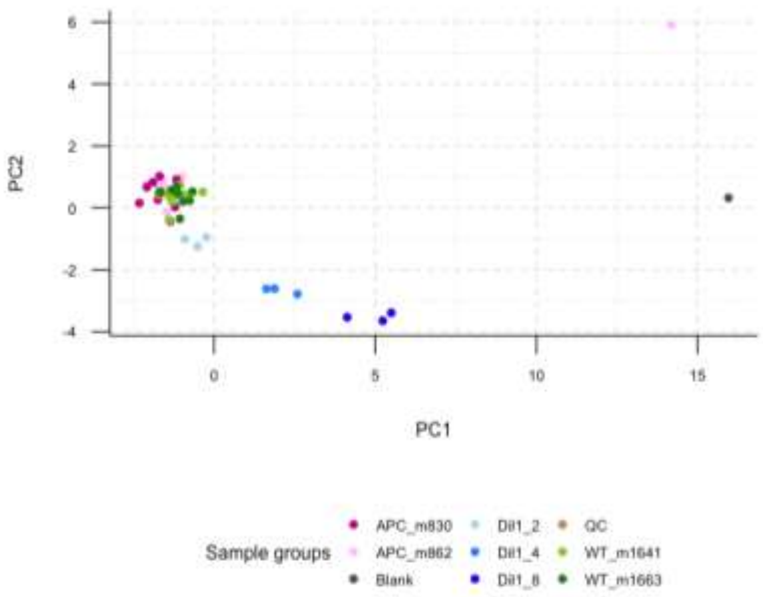

Figure S1. A PCA scores plot of LC-MS (ESI+ mode) profiles of intestinal organoids including experimental samples, blanks, quantity control samples (QCs) and QC dilution series (1:2, 1:4, 1:8; V_{QC}:$V_{solvent}$). The plot shows a good separation of the blank and dilution series from the experimental samples. One outlier (APC_m862) is shown.

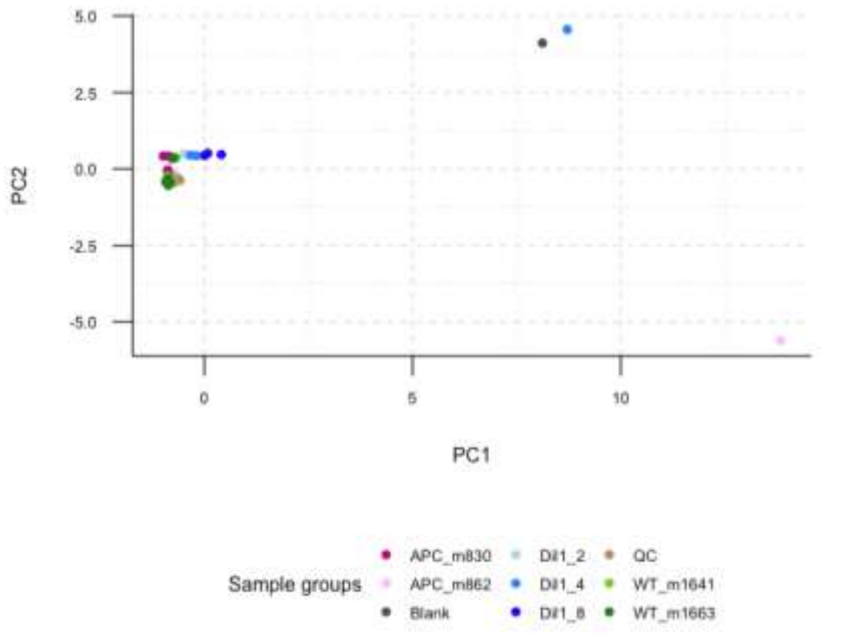

Figure S2. A PCA scores plot of LC-MS (ESI-mode) profiles of intestinal organoids including experimental samples, blanks, quantity control samples (QCs) and QC dilution series (1:2, 1:4, 1:8; V_{QC}:$V_{solvent}$). The plot shows a good separation of the blank and dilution series from the experimental samples. One outlier (APC_m862) is shown.

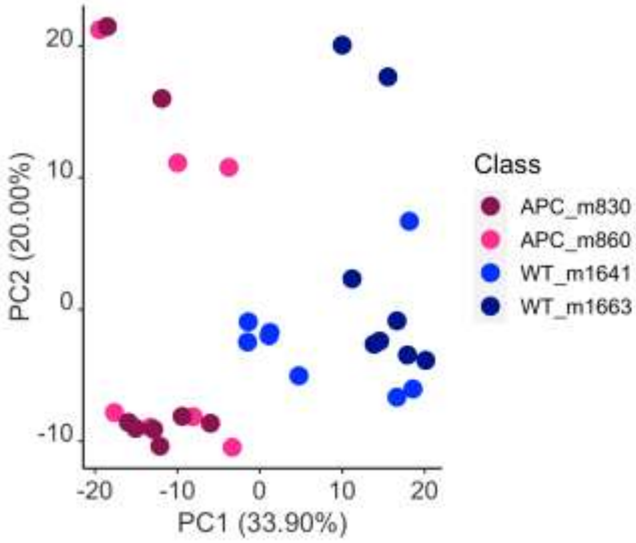

Figure S3.A PCA scores plot of LC-MS-based lipid profiles from *Apc*$^{fl/fl}$(pink) and WT (blue) organoids in ESI- (B) mode. The percentages on each axis represents proportions of variance (R^2X) explained by the principal component one and two (PC1 and PC2). Two shades of pink and blue colors represent the mouse donors of the organoids.

Table S1. Overview of LC-MS features that were found to be discriminant between wild type and *Apc*$^{fl/fl}$organoids (adjusted p-value < 0.05 and a VIP score > 1.5). RT: retention time.

APC vs WT (ESI+)		APC vs WT (ESI+)	
**RT (min)**	**Median m/z**	**RT (min)**	**Median m/z**
3.0	441.3552	13.1	628.6017
4.6	804.5730	13.1	970.7157
5.1	744.5700	13.1	822.6239
5.1	811.5542	13.1	1030.7393
5.1	902.6167	13.1	767.6579
5.6	846.5222	13.1	838.6644
5.6	636.4965	13.2	832.6627
5.7	795.5492	13.3	834.6891
5.8	666.483	13.3	684.6496
5.8	726.5598	13.3	808.662
5.8	794.5468	13.4	878.7032
5.8	667.4896	13.4	694.6695
5.8	884.6061	13.4	679.5839
5.8	844.6139	13.4	798.6302
6.0	810.6183	13.4	820.6637
6.2	789.5802	13.4	871.7216
6.2	766.5943	13.5	537.5243
6.3	723.5566	13.5	577.5173
6.3	683.5647	13.5	339.2893
6.4	886.6212	13.5	563.5384
7.2	741.5594	13.6	602.5838
7.4	768.5932	13.5	639.5921
7.7	744.5894	13.5	679.5824
8.0	1523.1707	13.5	706.603
8.3	884.595	13.5	818.6657
8.5	750.5833	13.6	580.6028
8.6	820.6415	13.6	603.5876
9.0	958.6784	13.6	654.6218
9.3	720.5893	13.6	860.6917
9.5	748.6118	13.6	998.748
9.6	862.5904	13.7	837.6801
9.9	862.5886	13.7	795.6891
10.2	728.5588	13.7	706.6305
10.3	728.5586	13.7	630.618
11.0	823.6599	13.7	787.6245
11.0	845.6422	13.7	1042.7736
11.9	1012.7257	13.8	694.6695
11.9	986.7106	13.8	836.6935
12.4	864.6078	13.8	717.6551
12.5	780.631	13.9	668.655
12.7	796.618	13.9	707.6149
12.8	549.4871	13.9	984.7693
12.8	1525.9305	13.9	313.2738
12.8	512.1979	14.0	707.6144
12.8	843.6919	14.0	898.7239
12.8	677.5681	14.1	608.6338
12.9	1379.8749	14.1	698.6423
12.9	652.5539	14.1	658.646
12.9	651.5509	14.1	656.6311
12.9	792.6366	14.1	614.6243
12.9	574.552	14.2	698.6415
12.9	552.571	14.3	864.7245

APC vs WT (ESI-)	
**RT (min)**	**Median m/z**
5.8	747.5986
7.1	1176.825
7.1	747.6105
8.1	572.5068
8.3	838.6171
12.1	1008.72
12.9	1356.8838
12.9	1402.8827
13.1	854.6718
13.1	829.6636
13.2	1036.7493
13.3	890.6925
13.4	900.748
13.7	810.6819
13.7	1064.7855
13.8	858.733
13.8	858.7454
13.9	856.756
13.8	848.6732
13.8	848.7046

WT (m1641 vs m1663 (ESI-)	
**RT min**	**median m/z**
12.8	889.6726
13.8	858.7454
13.8	669.6255
12.8	862.6565
12.8	889.7044
12.8	889.6785
14.1	684.6002
13.8	669.6142
13.6	656.5942

13.0	677.5684	14.3	676.6485
13.0	907.6201	14.3	653.6634
13.0	754.5711	14.5	658.6463
13.0	593.5779	14.5	900.74
13.0	591.5713	14.5	700.6571
13.0	578.5858	15.6	941.8126
13.0	600.5686	15.6	915.7977
13.1	872.6706	15.9	969.8443
13.1	704.6149	15.9	943.8284
13.1	792.6692	16.1	971.86
13.1	668.5963		
